# Prognostic value of admission blood glucose level in patients with and without diabetes mellitus who sustain ST segment elevation myocardial infarction complicated by cardiogenic shock

**DOI:** 10.1186/cc13035

**Published:** 2013-10-03

**Authors:** Jeong Hoon Yang, Pil Sang Song, Young Bin Song, Joo-Yong Hahn, Seung-Hyuk Choi, Jin-Ho Choi, Sang Hoon Lee, Myung-Ho Jeong, Young-Jo Kim, Hyeon-Cheol Gwon

**Affiliations:** 1 Division of Cardiology, Department of Medicine, Samsung Medical Center, Sungkyunkwan University School of Medicine, 50 Irwon-dong, Gangnam-gu, Seoul 135-710, Korea; 2 Department of Critical Care Medicine, Samsung Medical Center, Sungkyunkwan University School of Medicine, 50 Irwon-dong, Gangnam-gu, Seoul 135-710, Korea; 3 Department of Medicine, Inje University College of Medicine, Haeundae Paik Hospital, 1435 Jwa-dong, Haeundae-gu, Busan 612-030, Korea; 4 Division of Cardiology, Department of Medicine, Chonnam National University, 42 Jebong-ro, Dong-gu, Gwangju 501-757, Korea; 5 Division of Cardiology, Department of Medicine, Yeungnam University Hospital, 170 Hyeonchungno, Nam-gu, Daegu 705-717, Korea

## Abstract

**Introduction:**

Admission blood glucose (BG) level is a predictor of mortality in patients with ST-segment elevation myocardial infarction (STEMI). However, limited data are available relating admission BG to mortality in patients with STEMI complicated by cardiogenic shock, and it is not known whether diabetic status has an independent effect on this relationship.

**Methods:**

Between November 2005 and September 2010, 816 STEMI patients with cardiogenic shock were enrolled in a nationwide, prospective, multi-center registry; 239 (29.3%) had diabetes mellitus (DM). Patients were categorized according to BG levels at admission: <7.8, 7.8–10.9, 11.0–16.5 and ≥ 16.6 mmol/L. The primary outcome was 30-day mortality. The added values of BG to the Thrombolysis in Myocardial Infarction (TIMI) and Global Registry of Acute Coronary Events (GRACE) scores were assessed by receiver operating characteristic curves and integrated discrimination improvement analyses.

**Results:**

Thirty-day mortality was higher in patients with higher admission BG (20.4%, 23.3%, 39.8%, and 43.1% *p* < 0.001). Among non-diabetic patients, 30-day mortality was predicted by TIMI scores with a *c*-statistic of 0.615 (95% confidence interval [CI], 0.561–0.662) and GRACE scores with a *c*-statistic of 0.652 (95% CI, 0.604–0.695). Incorporation of admission BG increased the *c*-statistic for TIMI score to 0.685 (95% CI, 0.639–0.720, *p* < 0.001) and GRACE score to 0.708 (95% CI 0.664–0.742, *p* < 0.001). Additional predictive values for BG were not observed for diabetes. Integrated discrimination improvements (TIMI vs. additional BG and GRACE vs. additional BG) were 0.041 (*p* < 0.001) and 0.039 (*p* < 0.001) in non-diabetic patients.

**Conclusions:**

In a cohort of patients with STEMI complicated by cardiogenic shock, admission BG was an independent predictor of increased risk of mortality only among patients without DM.

## Introduction

Cardiogenic shock complicating ST-segment elevation myocardial infarction (STEMI) remains a leading cause of death with a hospital mortality rate approaching 50% [1]. The identification of risk predictors of mortality is important for tailoring more aggressive therapies that can improve survival in patients with cardiogenic shock [2]. Admission blood glucose (BG) level is an independent predictor of mortality in patients with STEMI, regardless of diabetic status and the occurrence of the no-reflow phenomenon [3-6]. In addition, hyperglycemia is associated with a higher level of cardiac necrosis markers [7]. Elevated BG level is proposed to be caused by a complex interplay between counteracting regulatory hormones such as cortisol, glucagon, growth hormone, and cytokines [8,9]. In heterogeneous populations of critically ill patients, hyperglycemia is independently associated with mortality [10,11]. However, limited data are available for stratified analysis of the presence or absence of diabetes mellitus (DM) in patients with cardiogenic shock. We therefore investigated the impact of admission BG level on 30-day mortality among patients with STEMI complicated by cardiogenic shock. Furthermore, to improve the effectiveness of admission BG level as a marker for short-term mortality, we evaluated the additive predictive value of BG level at admission to the Thrombolysis in Myocardial Infarction (TIMI) and Global Registry of Acute Coronary Events (GRACE) risk scores according to the presence or absence of DM.

## Materials and methods

### Study population

**Figure 1:**
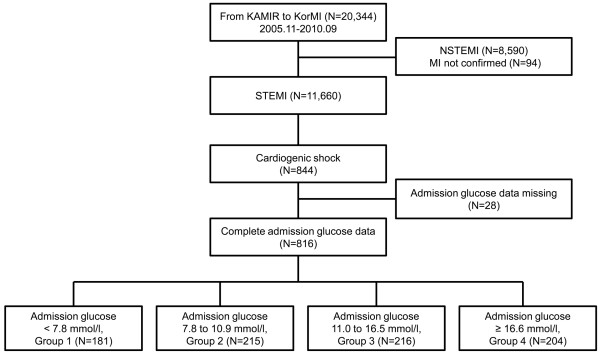
**Scheme of group distribution in the registry.** KAMIR, Korea Acute Myocardial Infarction Registry; KorMI, Korea Working Group on Myocardial Infarction Registry; NSTEMI, non-ST-segment elevation myocardial infarction; STEMI, ST-segment elevation myocardial infarction.

The study population was selected from the Korea Acute Myocardial Infarction Registry between November 2005 and December 2007 and from the registry series Korea Working Group on Myocardial Infarction Registry between January 2008 and September 2010. Data are from a prospective multicenter, online registry in Korea, with 53 hospitals registering consecutive patients with acute myocardial infarction. Between November 2005 and September 2010, 20,344 patients were enrolled in the registries. Data were collected by trained study coordinators using a standardized case report form and protocol. This registry was sponsored by the Korean Society of Cardiology. The institutional review board of all participating centers approved the study protocol (see Appendix). All participating patients provided written informed consent. Inclusion criteria were: consecutive patients 18 years of age or older; ST-segment elevation of 1 mm or more in two or more contiguous leads and new left bundle-branch block with at least one positive cardiac biochemical marker of necrosis; and diagnosis of cardiogenic shock. Exclusion criteria were: unavailable BG level at admission; and mechanical complications such as ventricular septal defect or mitral regurgitation from myocardial infarction. Patients were stratified according to BG levels at admission into group 1 (<7.8 mmol/l; *n* = 181), group 2 (7.8 to 10.9 mmol/l; *n* = 215), group 3 (11.0 to 16.5 mmol/l; *n* = 216) or group 4 (≥16.6 mmol/l; *n* = 204) (Figure 1). Patients with a previous history of DM treated with insulin, oral antihyperglycemic agents, or lifestyle modification at index hospital admission were classified as patients with DM. The patient flow of the study is shown in Figure 1. Clinical, laboratory, and outcome data were collected using a Web-based reporting system. Additional information was documented by contacting general practitioners and by review of hospital records. The primary outcome was 30-day mortality.

### Definitions

A clinical diagnosis of cardiogenic shock was made if all of the following were present: systolic blood pressure persistently lower than 90 mmHg or vasopressors required to maintain a blood pressure greater than 90 mmHg; signs of hypoperfusion (for example, urine output less than 30 ml/hour or cold/diaphoretic extremities or an altered mental status); and clinical evidence of left ventricular filling pressure (for example, pulmonary congestion on physical examination or chest X-ray scan). Vasopressor use was defined as use of dopamine, norepinephrine, and epinephrine regardless of their minimal dose. Thirty-day mortality was defined as any death during or after the procedure within 30 days and was considered to be of cardiac origin unless documentation indicated another cause. Admission BG was obtained by arterial or venous samples in an emergency room and measured with an arterial blood gas analyzer or laboratory analyzers in routine use at each hospital.

### Calculation of the TIMI and GRACE risk scores

Clinical scoring included the TIMI and GRACE risk scores for all enrolled patients. Full details of the design and methods of the TIMI and GRACE risk scores have been previously published [12,13]. The TIMI risk score consists of age, prior angina, diabetes, hypertension, systolic blood pressure <100 mmHg, heart rate >100 beats/minute, Killip class II to IV, weight <67 kg, anterior ST-segment elevation or left bundle branch block on electrocardiogram, and time to thrombolytics >4 hours. The GRACE risk score consists of age, heart rate, systolic blood pressure, serum creatinine level at presentation, Killip class, cardiac arrest at admission, ST-segment deviation on electrocardiogram, and elevated cardiac enzymes. Values for these variables were entered into the GRACE risk calculator [14] to obtain estimates of the cumulative risks of all-cause mortality.

### Statistical analysis

All continuous values were presented as the mean with standard deviation or the median with interquartile range. Categorical variables were presented as frequencies and percentages. Comparisons between continuous variables were tested using analysis of variance or the Kruskal–Wallis test, as appropriate. Categorical data were tested using the chi-squared test or Fisher’s exact test, as appropriate. Event-free survival was estimated by the Kaplan–Meier method and compared using the log-rank test within 30 days. Covariates statistically significant on univariate analysis (*P* <0.05) and those clinically relevant were considered candidate variables in multivariate models. Cox proportional-hazards models were adjusted by covariates as follows: age >75 years, sex, body weight <67 kg, systolic blood pressure (per 10 mmHg increase), anterior wall infarction on electrocardiography, serum creatinine >1.5 mg/dl, mechanical ventilation, intra-aortic balloon pump and admission BG level (group). Binary logistic regression was employed to measure the predictive probabilities of the combination of BG level and established risk score models for 30-day mortality. Comparisons among the various risk models of the predictive accuracy with the *c*-statistic, a measure of the area under the receiver-operator characteristic curve, were carried out using the procedure proposed by DeLong and colleagues according to the presence or absence of DM [15]. The 95% confidence intervals (CIs) were calculated using the bootstrapping procedure (*n* = 1,000 bootstrap samples) for area under the receiver-operator characteristic curve estimations and the differences between model areas under the receiver-operator characteristic curve. Furthermore, we estimated the integrated discrimination improvement to examine whether predictions based on the TIMI and GRACE risk models without admission BG level were significantly improved after inclusion of the admission BG level as a continuous parameter [16]. Statistical analyses were performed with SAS 9.2 (SAS Institute Inc., Cary, NC, USA) or Stata version 11.0 (StataCorp, College Station, TX, USA). All tests were two tailed and P <0.05 was considered statistically significant.

## Results

### Baseline and procedural characteristics

Between November 2005 and September 2010, 844 patients presented with STEMI complicated by cardiogenic shock. Of these, 816 (96.7%) had complete admission BG data (Figure 1). The mean admission BG level was 13.2 ± 7.0 mmol/l. Baseline clinical characteristics are presented in Table 1; 239 patients (29.3%) were classified as having DM, including 21 patients treated with insulin. Overall, patients with higher admission BG levels were higher-risk subjects. This group had a higher prevalence of females, DM, low systolic and diastolic blood pressure, anterior wall myocardial infarction, and elevated serum creatinine and TIMI risk score. Treatment during hospitalization is shown in Table 2; 644 (78.9%) underwent primary percutaneous coronary intervention. Similarly, patients with higher admission BG levels had higher use of mechanical ventilation and intra-aortic balloon pumps, and were less likely to receive aspirin, clopidogrel, and statins.

**Table 1:** Baseline patient characteristics

	**Serum glucose level on admission**				
**Variable**	**<7.8 mmol/l**	**7.8 to 10.9 mmol/l**	**11.0 to 16.5 mmol/l**	**≥16.6 mmol/l**	***P *** **value**
	**(group 1, *n *** **= 181)**	**(group 2, *n *** **= 215)**	**(group 3, *n *** **= 216)**	**(group 4, *n *** **= 204)**	
Age (years)	67.0 (56.0 to 76.0)	67.0 (56.0 to 76.0)	68.0 (56.0 to 76.0)	67.0 (58.0 to 75.0)	0.804
Sex (male)	126 (69.6)	144 (67.0)	126 (58.3)	117 (57.4)	0.022
Body weight (kg)	61.0 (53.0 to 70.0)	61.0 (55.0 to 69.5)	61.0 (55.0 to 69.3)	61.5 (55.0 to 69.0)	0.766
Diabetes					<0.001
Insulin	1 (0.6)	2 (0.9)	8 (3.7)	10 (4.9)	
Oral hypoglycemic agent	23 (12.7)	21 (9.8)	52 (24.1)	85 (41.7)	
Life style modification	1 (0.6)	2 (0.9)	11 (5.1)	17 (8.3)	
Hypertension	82 (45.3)	105 (48.8)	122 (56.5)	108 (52.9)	0.130
Dyslipidemia	51 (28.2)	49 (22.8)	39 (18.1)	39 (19.1)	0.070
Current smoker	88 (26.3)	91 (27.2)	82 (24.5)	74 (22.1)	0.066
Family history of CAD	13 (7.2)	15 (7.0)	16 (7.4)	15 (7.4)	0.998
Previous PCI	9 (5.0)	13 (6.1)	7 (3.2)	8 (3.9)	0.526
Previous CABG	2 (1.1)	1 (0.5)	3 (1.6)	0	0.329
Systolic blood pressure (mmHg)	90 (75 to 120)	80 (70 to 104)	80 (60 to 110)	75 (50 to 100)	<0.001
Diastolic blood pressure (mmHg)	60 (50 to 71)	51 (40 to 70)	50 (40 to 70)	50 (34 to 60)	<0.001
Heart rate (beats/minute)	74 (55 to 95)	66 (46 to 89)	68 (48 to 100)	68 (37 to 104)	0.116
Infarct location on electrocardiogram					
Anterior or LBBB	84 (46.4)	87 (40.5)	112 (51.9)	110 (53.9)	0.027
Inferior	76 (42.0)	113 (52.6)	92 (42.6)	77 (37.7)	0.018
Other	21 (11.6)	15 (7.0)	12 (5.6)	17 (8.3)	0.151
Left ventricular ejection fraction	46.5 (39.0 to 57.0)	47.0 (39.0 to 56.0)	46.0 (36.0 to 57.0)	44.0 (35.0 to 52.5)	0.100
Serum creatinine (mg/dl)	1.1 (0.9 to 1.4)	1.1 (1.0 to 1.4)	1.2 (1.0 to 1.5)	1.3 (1.1 to 1.6)	<0.001
TIMI risk score	8 (6 to 10)	8 (6 to 10)	8 (7 to 10)	8 (7 to 10)	0.013
GRACE risk score	227.0 (205.0 to 245.0)	228.0 (208.0 to 248.0)	230.5 (210.0 to 250.3)	230.0 (216.8 to 248.5)	0.227

Values presented as mean ± standard deviation, median (interquartile range) or *n* (%).

Comparisons between continuous variables were all tested using the Kruskal–Wallis test. CABG, coronary artery bypass grafting; CAD, coronary artery disease; GRACE, Global Registry of Acute Coronary Events; LBBB, left bundle branch block; PCI, percutaneous coronary intervention; TIMI, Thrombolysis in Myocardial Infarction.

**Table 2:** Treatment during hospitalization

**Variable**	**Serum glucose level on admission**				
	**<7.8 mmol/l**	**7.8 to 10.9 mmol/l**	**11.0 to 16.5 mmol/l**	**≥16.6 mmol/l**	***P *** **value**
	**(group 1, *n *** **= 181)**	**(group 2, *n *** **= 215)**	**(group 3, *n *** **= 216)**	**(group 4, *n *** **= 204)**	
Primary PCI	138 (76.2)	177 (82.3)	167 (77.3)	162 (79.4)	0.450
Facilitated PCI	6 (3.3)	3 (1.4)	3 (1.4)	2 (1.0)	0.380
Thrombolysis	11 (6.1)	8 (3.7)	9 (4.2)	6 (2.9)	0.470
Medical treatment	26 (14.4)	27 (12.6)	37 (17.1)	34 (16.7)	0.527
Aspirin	175 (96.7)	203 (94.4)	205 (94.9)	177 (86.8)	<0.001
Clopidogrel	173 (95.6)	205 (95.3)	200 (92.6)	176 (86.3)	0.001
Low molecular weight heparin	48 (26.5)	56 (26.0)	53 (24.5)	47 (23.0)	0.852
Glycoprotein IIb/IIIa inhibitor	23 (12.7)	32 (14.9)	42 (19.4)	42 (20.6)	0.121
Beta-blockers	96 (53.0)	123 (57.2)	100 (46.3)	104 (51.0)	0.151
ACE inhibitors	92 (50.8)	107 (49.8)	101 (46.8)	95 (46.6)	0.778
Angiotensin receptor blocker	27 (14.9)	31 (14.4)	27 (12.5)	28 (13.7)	0.905
Statins	119 (65.7)	136 (63.3)	118 (54.6)	107 (52.5)	0.016
Mechanical ventilation	48 (26.5)	49 (22.8)	86 (39.8)	85 (41.7)	<0.001
Intra-aortic balloon pump	38 (21.0)	46 (21.4)	66 (30.6)	63 (30.9)	0.023
Defibrillator/cardioversion	32 (17.7)	38 (17.7)	46 (21.3)	50 (24.5)	0.257
Temporary pacemaker	22 (12.2)	33 (15.3)	42 (19.4)	40 (19.6)	0.148

Values presented as *n* (%). ACE, angiotensin converting enzyme; PCI, percutaneous coronary intervention.

### Clinical outcomes

**Figure 2:**
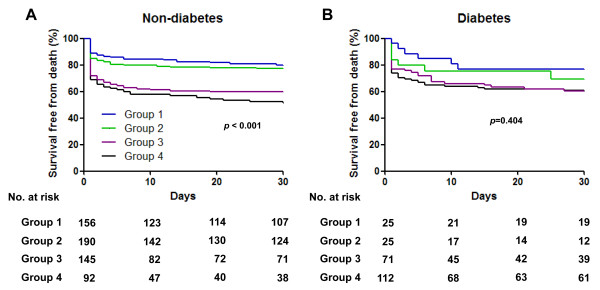
**Unadjusted Kaplan–Meier curves.** Survival based on admission glucose level in **(A)** patients without diabetes and **(B)** patients with diabetes.

Figure 2 shows the cumulative 30-day mortality of the study population stratified by admission BG level and presence or absence of diabetes. Total 30-day mortality was 32.0%; 30-day mortality in patients with diabetes was 36.0% compared with 30.3% for patients without diabetes (*P* = 0.115). In the total study population, 30-day mortality rates were higher in patients with higher admission BG levels (20.4% for patients with admission BG level <7.8 mmol/l, 23.3% for 7.8 to 10.9 mmol/l, 39.8% for 11.0 to 16.5 mmol/l, and 43.1% for ≥16.6 mmol/l, *P* <0.001). Similarly, among nondiabetic patients, 30-day mortality rates were higher in patients with higher admission BG levels (20.0%, 22.6%, 40.1%, and 48.9%, *P* <0.001). However, diabetic patients showed no significant difference in 30-day mortality according to admission BG level (23.1%, 28.0%, 39.2%, and 38.6%, *P* = 0.404).

### Predictors of 30-day mortality in patients with and without diabetes

Cox regression analysis was performed to determine predictors of 30-day mortality (Table 3). In nondiabetic patients, independent predictors for the occurrence of 30-day mortality were female, anterior wall myocardial infarction, serum creatinine >1.5 mg/dl, mechanical ventilation support, use of intra-aortic balloon pump, and admission BG level group. In diabetic patients, the independent predictors for the occurrence of 30-day mortality were age ≥75 years, female, and low systolic blood pressure.

**Table 3:** Significant predictors of 30-day mortality with and without diabetes

	**Nondiabetes (*n *** **= 577)**		**Diabetes (*n *** **= 239)**	
**Variable**	**Hazard ratio**^**a **^**(97.5% CI)**	**Adjusted *P *** **value**	**Hazard ratio**^**a **^**(97.5% CI)**	**Adjusted *P *** **value**
Age ≥75 years	1.10 (0.74 to 1.56)	0.999	1.80 (1.00 to 3.23)	0.049
Sex (female)	1.93 (1.29 to 2.90)	<0.001	1.97 (1.07 to 3.62)	0.026
Body weight <67 kg	1.03 (0.67 to 1.60)	0.999	0.76 (0.39 to 1.46)	0.689
Systolic blood pressure, per 10 mmHg increase	0.96 (0.93 to 1.01)	0.108	0.94 (0.87 to 0.99)	0.044
Anterior wall infarction on electrocardiography	1.94 (1.34 to 2.82)	<0.001	1.47 (0.85 to 2.57)	0.235
Wide QRS tachycardia on admission	1.55 (0.95 to 2.53)	0.093	1.64 (0.69 to 3.91)	0.406
Serum creatinine >1.5 mg/dl	1.56 (1.09 to 2.25)	0.011	1.61 (0.92 to 2.81)	0.113
Mechanical ventilation	2.36 (1.59 to 3.48)	<0.001	1.81 (0.99 to 3.32)	0.056
Intra-aortic balloon pump	1.54 (1.06 to 2.23)	0.019	1.04 (0.57 to 1.87)	0.999
Admission glucose level (vs. group 1)				
Group 2 (7.8 to 10.9 mmol/l)	1.19 (0.69 to 2.06)	0.945	2.40 (0.62 to 9.31)	0.296
Group 3 (11.0 to 16.5 mmol/l)	1.76 (1.06 to 2.94)	0.026	1.74 (0.59 to 5.18)	0.507
Group 4 (≥16.6 mmol/l)	1.72 (1.02 to 3.00)	0.030	1.74 (0.60 to 5.04)	0.487

CI, confidence interval.

^a^Adjusted covariates include age >75 years, sex, body weight <67 kg, systolic blood pressure (per 10 mmHg increase), anterior wall infarction on electrocardiography, serum creatinine >1.5 mg/dl, mechanical ventilation, intra-aortic balloon pump and admission glucose level (group).

### Additive prognostic value of admission glucose level to TIMI and GRACE scores

**Figure 3:**
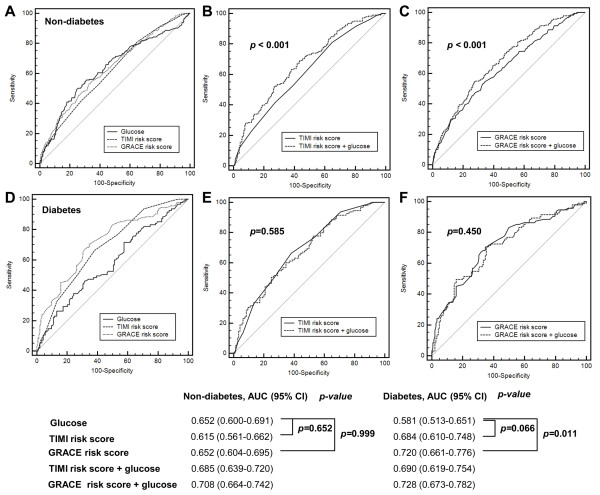
**Receiver operating characteristics analysis of predictors of 30-day mortality.** Comparison of area under receiver-operating characteristics curve (AUC) analysis between admission glucose level and the TIMI risk score or GRACE risk score among **(A)** nondiabetic and **(D)** diabetic patients. Comparing the discrimination of the TIMI risk score **(B)**, **(E)** and the GRACE risk score **(C)**, **(F)** alone with a model including admission glucose in predicting 30-day mortality according to the presence of diabetes mellitus. CI, confidence interval; TIMI, Thrombolysis in Myocardial Infarction; GRACE, Global Registry of Acute Coronary Events.

Receiver operating characteristic curves for admission BG level, TIMI risk score, GRACE risk score, and the combination of admission BG and TIMI or GRACE score are shown in Figure 3. Among nondiabetic patients, admission BG level and TIMI and GRACE scores predicted 30-day mortality, with *c*-statistics of 0.652 (95% CI, 0.600 to 0.691) for admission BG level, 0.615 (95% CI, 0.561 to 0.662) for TIMI score, and 0.652 (95% CI, 0.604 to 0.695) for GRACE score. The *c*-statistics scores increased significantly to 0.685 (95% CI, 0.639 to 0.720, *P* <0.001) for TIMI and to 0.708 (95% CI 0.664 to 0.742, *P* <0.001) for GRACE when the admission BG level was added. Additionally, improvements in discrimination were confirmed by the integrated discrimination improvement (0.041 (95% CI, 0.022 to 0.059), *P* <0.001 for TIMI vs. incorporation of BG into TIMI; and 0.039 (95% CI, 0.021 to 0.057), *P* <0.001 for GRACE vs. incorporation of BG into GRACE).

In contrast, in diabetic patients the admission BG level performed poorly, with a *c*-statistic of 0.581 (95% CI, 0.513 to 0.651), while the GRACE risk score predicted the occurrence of 30-day mortality with a *c*-statistic of 0.720 (95% CI, 0.661 to 0.776) (glucose vs. GRACE, *P* = 0.011). Performances were not improved by the addition of admission BG level to TIMI and GRACE risk score with a nonsignificant increase in the *c*-statistic of 0.06 for TIMI (*P* = 0.585) and 0.08 for GRACE score (*P =* 0.450). Furthermore, significant improvements in discrimination were not observed with the integrated discrimination improvement (0.012 (95% CI, 0.003 to 0.027), *P =* 0.104 for TIMI vs. incorporation of BG into the TIMI; or 0.014 (95% CI, 0.002 to 0.031), *P =* 0.084 for GRACE vs. incorporation of BG into GRACE).

## Discussion

We investigated the impact of admission BG level on 30-day mortality and evaluated the additional predictive value of admission BG to the TIMI and GRACE risk scores among patients with STEMI complicated by cardiogenic shock stratified by the presence or absence of DM. Subjects were enrolled from a nationwide, prospective, multicenter registry in Korea. The major findings of this study were that the 30-day mortality rates were higher in patients with higher admission BG levels in nondiabetic patients but not in diabetic patients, and that the admission BG level had an additional predictive value to TIMI and GRACE risk score models in nondiabetic patients but not in diabetic patients.

Acute hyperglycemia on admission and during hospital stay is associated with adverse outcome in patients with acute myocardial infarction regardless of diabetic status [7,9,17,18]. In cardiogenic shock, gluconeogenesis from acute stress could be more prominent. In our study, the mean admission BG level (13.2 mmol/l) was considerably higher than a previous study of STEMI patients with mainly no hemodynamic compromise (7.8 mmol/l). In patients with STEMI complicated by cardiogenic shock, our study found that higher BG levels at admission were associated with higher mortality in the nondiabetic population but not the diabetic population. Similarly, a recent study failed to find a significant relationship between hyperglycemia and mortality in diabetic patients with acute myocardial infarction [19]. In addition, Ishihara and colleagues described a different relationship between admission BG and in-hospital mortality in nondiabetic and diabetic patients, and a hyperglycemia-associated risk that was more obvious in nondiabetic patients than in patients with diabetes [20]. In a heterogeneous population of critically ill patients, mean BG is related to ICU mortality in the nondiabetic cohort but not in the diabetic cohort [21]. These findings suggest that the toxic effect of hyperglycemia on short-term mortality may be less in known diabetic patients. One possible explanation is that the definition and severity of stress hyperglycemia is difficult in diabetic patients because they are more likely to receive insulin or oral hypoglycemic agents before admission for myocardial infarction [22]. These treatments might mitigate the toxic effects of stress hyperglycemia and could disturb the BG level proportionate to the increase in acute stress in diabetic patients. Another possible explanation is that diabetes patients may develop a resistance to hyperglycemia, and a moderate or severe degree of hyperglycemia and glycemic variability that might exert toxicity in a patient without diabetes may be well tolerated in a patient with diabetes [10].

Identifying high-risk individuals with STEMI complicated by cardiogenic shock remains a challenge. The most widespread tool is the GRACE risk score, which was developed from a large unselected population of patients with all forms of acute coronary syndrome [13]. The TIMI risk score for STEMI is a simple alternative, derived from patients treated with fibrinolytics [12,23]. Although recent studies suggested variable risk-prediction models for cardiogenic shock, a robust risk model applicable to cardiogenic shock with wide range of mortality rates has not been developed [24,25]. In our study, incorporation of the admission BG level to the widely accepted TIMI and GRACE risk scores improved its predictive values. Moreover, admission BG level alone was comparable with the TIMI and GRACE risk scores for short-term mortality in nondiabetic patients. Accordingly, an addition of admission BG level to existing risk models could be helpful for developing a new risk model in cardiogenic shock and could improve initial bedside risk stratification.

Our study has several limitations. DM was defined as known diabetic status on admission. Many STEMI patients are known to have undetected DM, and they would not have been excluded in our study [7,17]. Major weakness of this study is the lack of identification data to distinguish nondiabetes from undetected diabetes. There is therefore no way to discern differential impact on mortality between true nondiabetes and undetected diabetes. We did not routinely measure glycosylated hemoglobin or test for diabetes during admission. Another limitation might be that although admission BG will be responsive to the acute stress associated with a STEMI, many other factors such as prior meals or diurnal variation can contribute to the variability of admission BG levels. Association of the higher admission BG with higher mortality within 30 days might continue in nondiabetic patients. However, the mechanism of this finding is unclear. In addition, we could not identify the impact of BG lowering therapy on mortality because data on BG levels during the course of hospitalization and therapeutic BG targets were not available in our registries. The initial treatment strategy on presentation of a high BG level was left to the operator’s discretion. Large-scale, randomized trials are needed to investigate a relationship between BG lowering therapy and mortality among patients with STEMI complicated by cardiogenic shock. Lastly, the low 30-day mortality of 32.0% in our trial might suggest that our investigation included a relatively high percentage of patients with mild or moderately severe cardiogenic shock. In fact, only 33% of the patients needed mechanical ventilation and 26% underwent insertion of an intra-aortic balloon pump.

## Conclusions

In STEMI complicated by cardiogenic shock, the admission BG level was independently associated with increased risk of 30-day mortality and had an additional predictive value for established risk score models in nondiabetic patients but not in diabetic patients.

## Key messages

•In nondiabetic patients with STEMI complicated by cardiogenic shock, 30-day mortality rates were higher in patients with higher admission BG levels.

•In diabetic patients with STEMI complicated by cardiogenic shock, there is no significant difference in 30-day mortality according to admission BG level.

•In a cohort of patients with STEMI complicated by cardiogenic shock, the admission BG level had an additional predictive value to TIMI and GRACE risk score models in nondiabetic patients but not in diabetic patients.

## Appendix

All institutional review boards that approved the study include Samsung Medical Center, Seoul National University Hospital, Gachon University Gil Hospital, Pusan National University Hospital, Chungnam National University Hospital, Chonbuk National University Hospital, Dong-A University Medical Center, Korea University Anam Hospital, Kosin University Gospel Hospital, Eulji Medical Center, Kyungpook National University Hospital, Inje University Busan Paik Hospital, Yeungnam University Medical Center, Korea University Guro Hospital, Seoul National University Bundang Hospital, Gyeongsang National University Hospital, Ewha Womans University Mokdong Hospital, Chonnam National University Hospital, Soon Chun Hyang University Hospital, Buncheon, Kyung Hee University Hospital, Wonkwang University Hospital, Konyang University Hospital, Wonju Christian Hospital, Hallym University Sacred Heart Hospital, Dankook University Hospital, Konkuk University Medical Center, Ulsan University Hospital, Daegu Catholic University Medical Center, Soon Chun Hyang University Hospital, Cheonan, Soon Chun Hyang University Hospital, Seoul, Chosun University Hospital, The Catholic University of Korea, Uijeongbu St. Mary’s Hospital, Inje University Sanggye Paik Hospital, Kangdong Sacred Heart Hospital, Chungbuk National University Hospital, Hanyang University Guri Hospital, Kyung Hee University Hospital at Gangdong, Korea University Ansan Hospital, St. Carollo Hospital, The Catholic Univerity of Korea, Daejeon St. Mary’s Hospital, Presbyterian Medical Center, Veterans Hospital, Dongguk University Ilsan Hospital, Chung-Ang University Hospital, Daejeon Sun Hospital, Jeju National University Hospital, Dongguk University Gyengju Hospital, Inje University Haeundae Paik Hospital, The Catholic University of Korea, St. Paul’s Hospital, SM Christianity Hospital, Kangwon National University Hospital, Best Hanseo Hospital, and Keimyung University Dongsan Medical Center.

## Abbreviations

BG: Blood glucose; DM: Diabetes mellitus; GRACE: Global registry of acute coronary events; STEMI: ST-segment elevation myocardial infarction; TIMI: Thrombolysis in myocardial infarction.

## Competing interests

The authors declare that they have no competing interests.

## Authors’ contributions

All authors contributed to the study design, acquisition of data, or analysis and interpretation of data, and have been involved in drafting the manuscript. Especially, JHY, PSS, YBS, J-YH, S-HC, J-HC, SHL, M-HJ, Y-JK, and H-CG participated in the enrollment of patients, performed the procedures, and contributed to clinical follow-up. JHY, PSS, YBS, J-YH, S-HC, J-HC, SHL, M-HJ, Y-JK, and H-CG participated in data collection. JHY, YBS, and H-CG participated in the data analysis. JHY, PSS, YBS, SHL, M-HJ, Y-JK, and H-CG contributed to data interpretation. JHY and H-CG contributed to writing of the manuscript. All authors gave final approval of the manuscript for publication.
